# Supplementary material for: Genetically engineered red cells expressing single domain camelid antibodies confer long-term protection against botulinum neurotoxin
Source: Nat Commun. 2017 Sep 4;8:423. doi: 10.1038/s41467-017-00448-0 (PMC5583347; doi:10.1038/s41467-017-00448-0)
Supplement: Supplementary file 1 — Supplementary Information [file 41467_2017_448_MOESM1_ESM.pdf]

File name: Supplementary Information
Description: Supplementary Figures

File name: Peer Review File
Description:

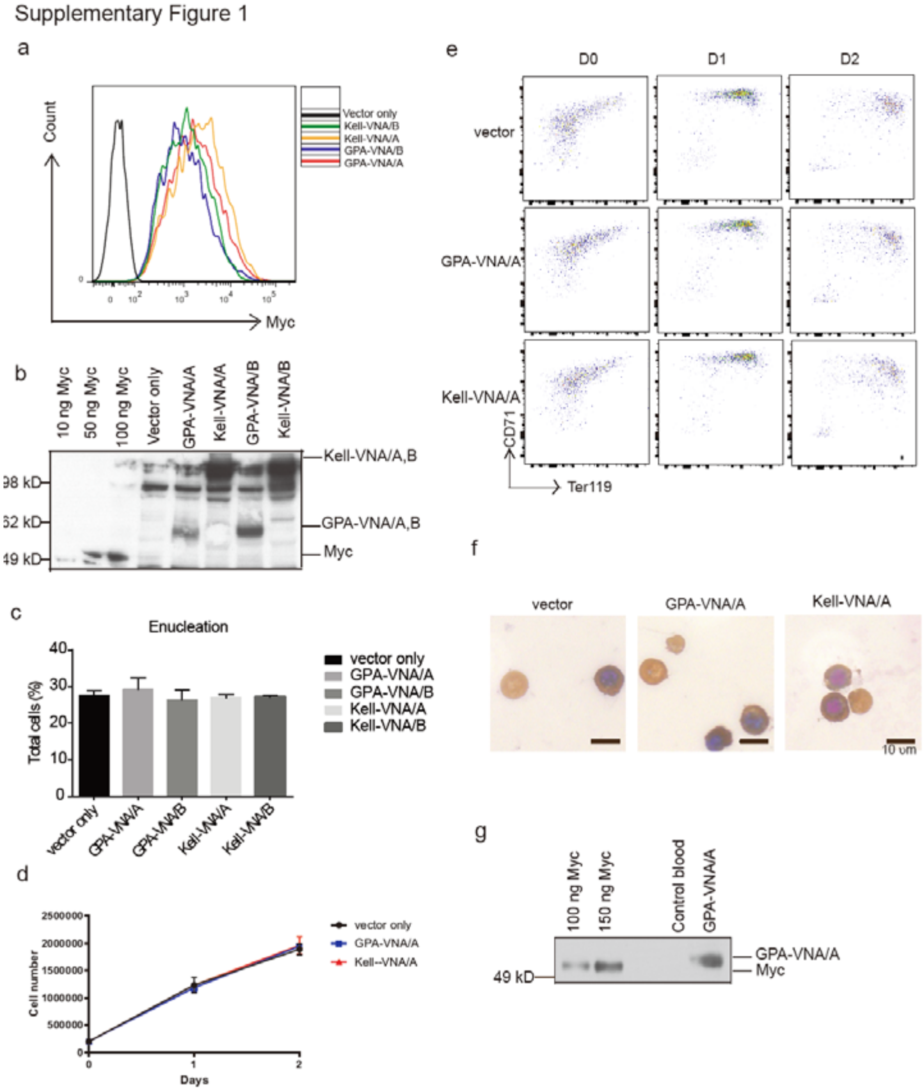

Supplementary Figure 1 Enucleation, proliferation and protein expression of engineered murine RBCs express chimeric GPA and Kell proteins.

In vitro culture - produced engineered RBCs were analyzed as followed: (a) Surface myc expression of murine RBCs that express empty vector, GPA-VNA/A, GPA-VNA/B, Kell-VNA/A or Kell-VNA/B was analyzed by FACS. (b) Murine RBCs expressing empty vector, GPA-VNA/A, GPA-VNA/B, Kell-VNA/A or Kell-VNA/B were produced by *in vitro* culture of fetal liver progenitors. Cell lysates of 1,000,000 RBCs and the

indicated amount of full-length recombinant protein with an attached myc epitope (molecular weight = 55 kD) were resolved in SDS-PAGE for western blotting. Western blots were performed with anti-Myc antibody. 10, 50, and 100 ng Myc proteins were used as a quantification standard. Each red cell expresses 4,600,000 GPA-VNA/A proteins and each red cell expresses 2,190,000 Kell-VNA/A proteins (see calculation in Materials and Methods section). (c) Enucleation of murine RBCs that express empty vector, GPA-VNA/A, GPA-VNA/B, Kell-VNA/A or Kell-VNA/B was quantified by flow cytometry. (n=3/group, mean+S.E.M.) (d) Proliferation curve of mouse red cells which were in vitro cultured to express vector, GPA-VNA/A and Kell-GPA/A. (n=3/group, mean±S.E.M.) (e) Surface expression of CD71 and Ter119 was examined by flow cytometry at the indicated culture time. (f) Giemsa and hemoglobin staining of mouse RBCs at the end of culture. (g) Expression of VNA/A on mature red blood cells produced in transplanted mice. Six million GFP+ red cells were sorted from the blood of chimeric mice previously transplanted with GPA-VNA/A - expressing progenitors. These GFP+ cells (GPA-VNA/A) and same number of control red cells were lysed and resolved by SDS-PAGE for western blotting performed with the same anti-myc antibody used in panel b. 100 and 150 ng full-length myc recombinant protein were used as an internal quantification standard. Calculated from the band's density, 6,000,000 GPA-VNA/A red cells express ~200 ng myc-GPA-VNA/A proteins, or about 310,000 VNA/A proteins per cell, about $1/15^{th}$ that found on red cells made in *in vitro* culture.

Supplementary Figure 2

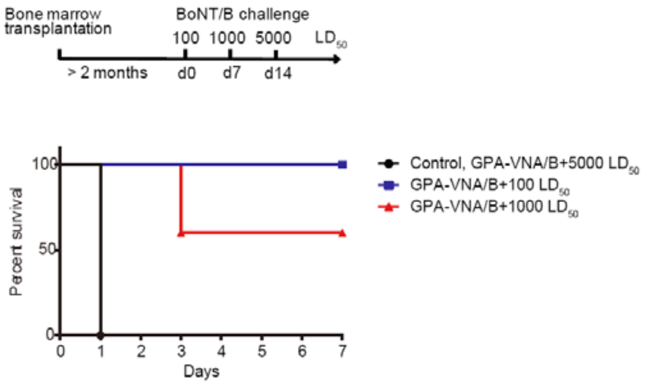

Supplementary Figure 2 Mice that produce murine RBCs carrying GPA-VNA/B are protected against BoNT/B challenge.

Survival curve of mice challenged with BoNT/B. Mice that were transplanted with stem cells/progenitors expressing GPA-VNA/B were challenged with 100 LD_{50} BoNT/B and surviving mice were challenged with 1000 LD_{50} the following week. After one week, a 5000LD_{50} BoNT/B were administrated to surviving mice. Mice were monitored for 7 days following each challenge (n=5/group).

Supplementary Figure 3

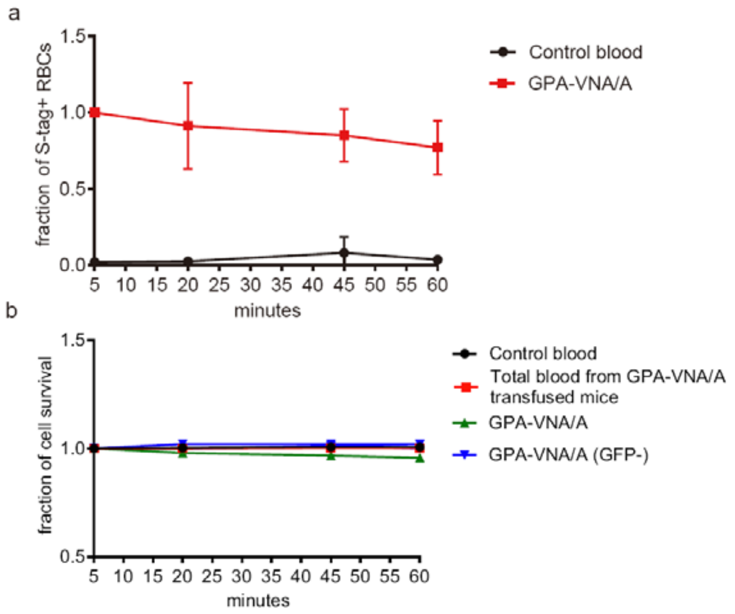

Supplementary Figure 3 Detection of ciBoNT/A and transfused RBCs 1 hour after transfusion

(a)(b) Detection of RBC-bound ciBoNT/A and transfused RBCs in the blood of recipient mice. Mice were treated as described in Fig. 2f and Fig. 2g. Recipients were bled at shorter time points as indicated and RBCs were subjected to flow cytometry analyses to quantify the S-tag (indirectly detecting RBC- bound ciBoNT/A, Panel a) as well as the violet-trace (total transfused RBCs, Panel b) (n=3/group, mean±S.E.M.).

Supplementary Figure 4

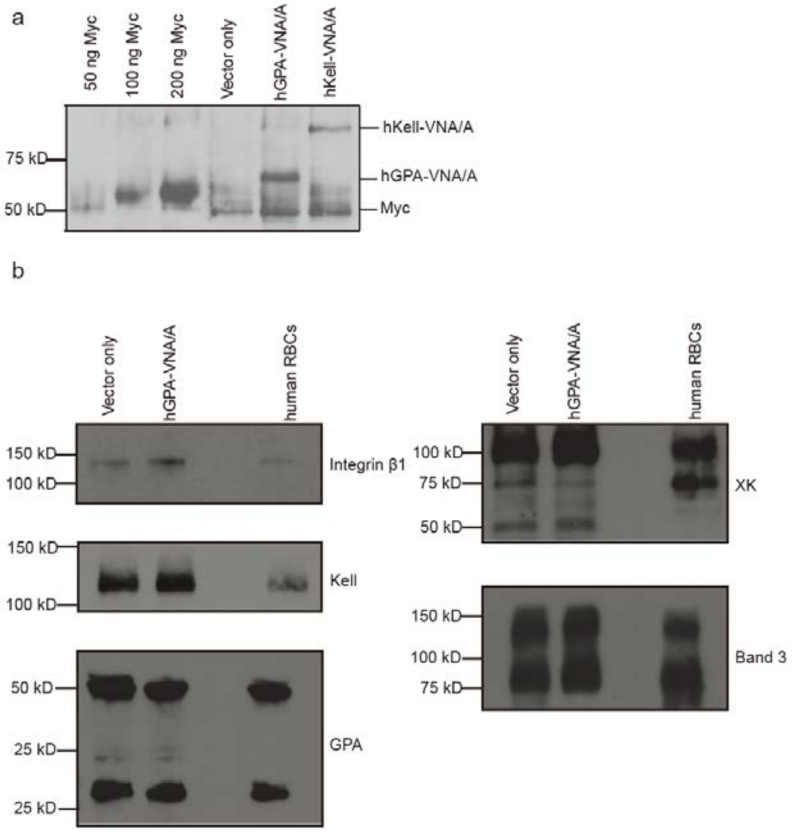

Supplementary Figure 4 (cont.)

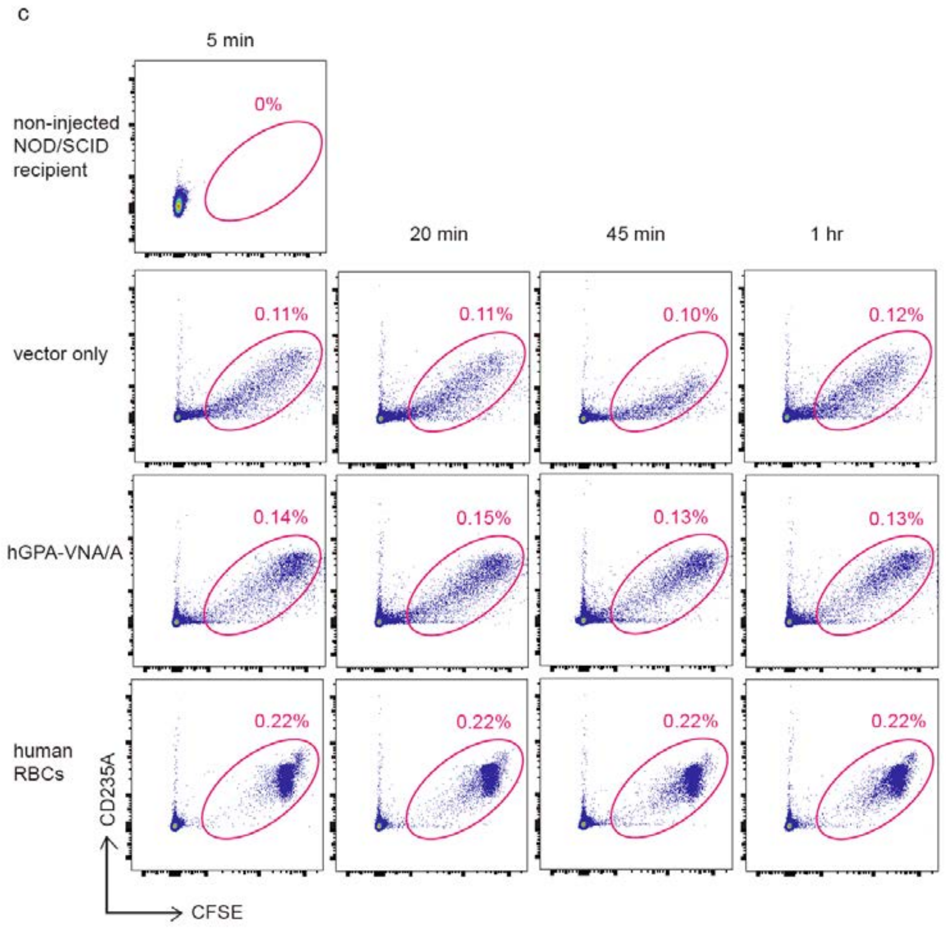

Supplementary Figure 4 Protein expression and in vivo survival of engineered human RBCs express chimeric GPA and Kell proteins.

(a) Human RBCs expressing empty vector, GPA-VNA/A or Kell-VNA/A were generated by *in vitro* culture. Cell lysates of 1,800,000 RBCs and full-length myc recombinant protein were resolved in SDS-PAGE. Western blot was performed with anti-myc antibody. 50, 100 and 200 ng full-length myc proteins were used as quantification standard. As determined by the myc signal intensity in the Western blot with an anti-myc antibody and as quantified by ImageJ, 1,800,000 human GPA-VNA/A cells contained

100 ng GPA-VNA/A proteins. By calculations similar to those in Figure S1, each human hGA-VNA/A red cell expresses 511,000 copies of GPA-VNA/A. Similarly, 1,800,000 Kell-VNA/A cells contained 40 ng Kell-VNA/A protein, equal to 121,000 copies of Kell-VNA/A protein per cell.

(b) Western blot of several membrane and cytoskeleton proteins from normal human red blood cells and in vitro cultured CD34+ cells expressing vector alone or GPA-VNA/A. (c) Circulation of cultured RBCs expressing vector, GPA-VNA/A or human RBCs in macrophage-depleted NOD/SCID mice. 100 million six stage- in vitro cultured RBCs and normal human RBCs were labeled with CFSE and injected intravenously into NOD/SCID mice that have been treated with clodronate liposomes. The recipient mice were then bled at the indicated time points and subjected to flow cytometry analyses. The transfused human RBCs were identified by CFSE and CD235A expression.

Supplementary Figure 5

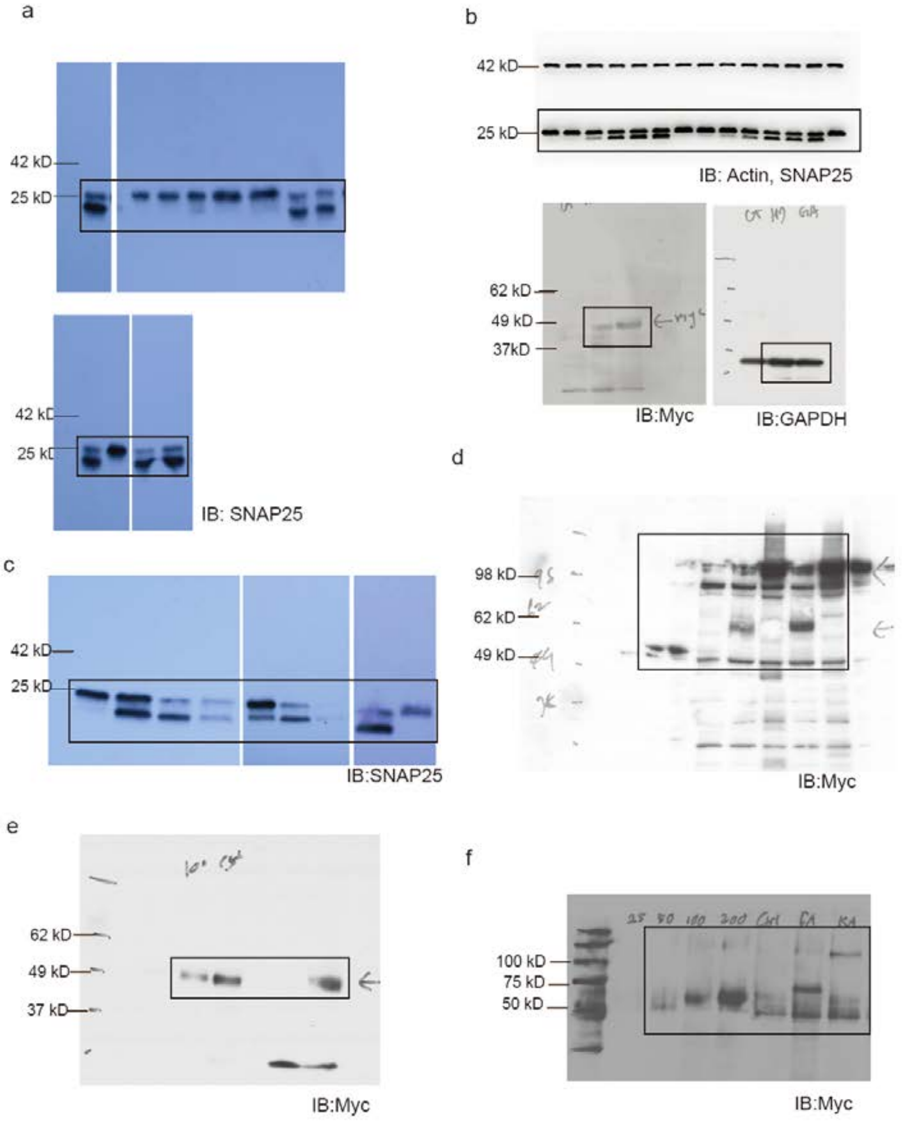

Supplementary Figure 5 (cont.)

g

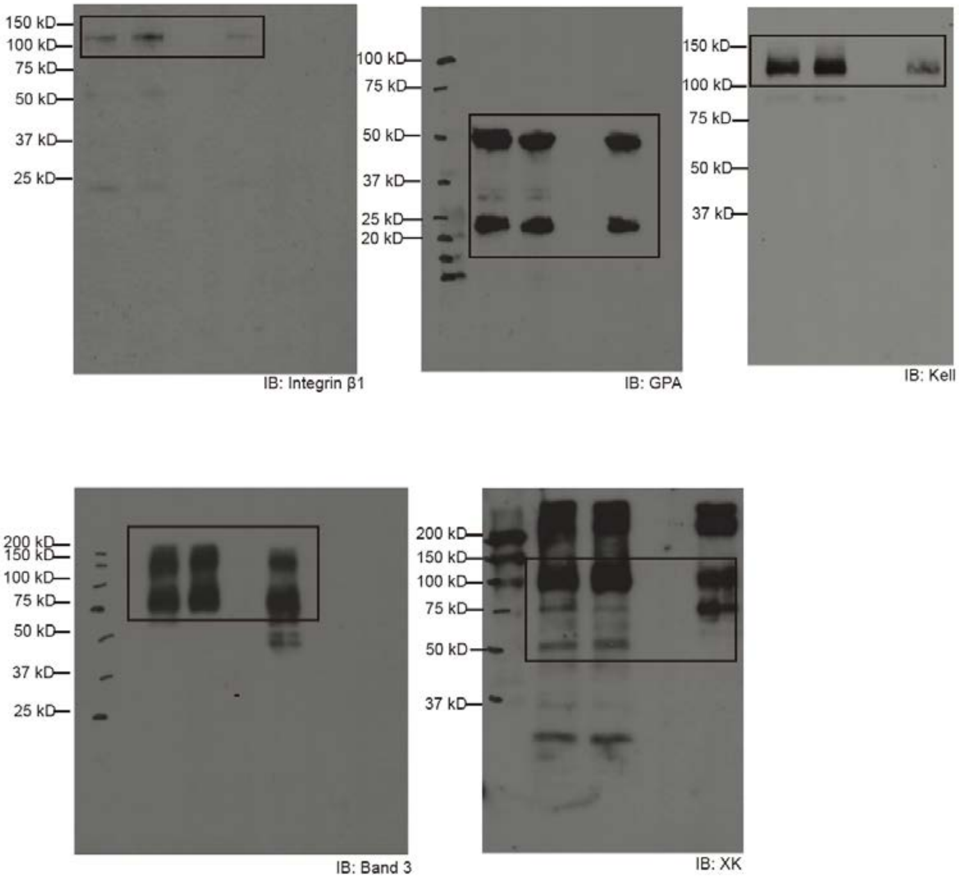

Supplementary Figure 5 Uncropped blot images

(a) Figure 1b. (b) Figure 3b. (c) Figure 5d. (d) Supplementary Figure 1b. (e) Supplementary Figure 1g. (f) Supplementary Figure 4a. (g) Supplementary Figure 4b.
